# Dihydrotestosterone induces arterial stiffening in female mice

**DOI:** 10.1186/s13293-024-00586-3

**Published:** 2024-01-23

**Authors:** Alec C. Horton, Mary M. Wilkinson, Isabella Kilanowski-Doroh, Zhejun Dong, Jiao Liu, Benard O. Ogola, Bruna Visniauskas, Sarah H. Lindsey

**Affiliations:** 1 Department of Pharmacology and Tulane Brain Institute, Tulane School of Medicine, New Orleans, LA USA; 2 Department of Pharmacology, Tulane School of Medicine, New Orleans, LA USA; 3 Department of Pediatrics, Tulane School of Medicine, Hayward Genetics Center, New Orleans, LA USA; 4 Vascular Biology Center and Department of Medicine, Medical College of Georgia at Augusta University, Augusta, GA USA; 5 Tulane Center of Excellence in Sex-Based Biology and Medicine, New Orleans, LA USA

**Keywords:** Testosterone, Arterial stiffness, Gender-affirming therapy, Hormones, Estrogen receptors, Polycystic ovarian syndrome

## Abstract

**Background:**

Androgens are important sex hormones in both men and women and are supplemented when endogenous levels are low, for gender transitioning, or to increase libido. Androgens also circulate at higher levels in women with polycystic ovarian syndrome, a condition that increases the risk for cardiovascular diseases including hypertension and arterial stiffness. Since our previous work shows an important role for the G protein-coupled estrogen receptor (GPER) in arterial stiffness, we hypothesized that other hormones including androgens may impact arterial stiffness in female mice via downregulation of GPER.

**Methods:**

The impact of the non-aromatizable androgen dihydrotestosterone (DHT), the glucocorticoid dexamethasone, and the progestin medroxyprogesterone acetate (all 100 nM for 24 h) on GPER and ERα expression was assessed in cultured vascular smooth muscle cells using droplet digital PCR (ddPCR)*.* To assess the in vivo impact of the DHT-induced downregulation of GPER, female ovary-intact C57Bl/6 mice at 15–16 weeks of age were treated with silastic capsules containing DHT for 4 weeks, one with a dosage expected to mimic human male DHT levels and another to double the expected human concentration (n = 8–9/group).

**Results:**

In cultured vascular smooth muscle cells, GPER mRNA was decreased by DHT (P = 0.001) but was not impacted by dexamethasone or medroxyprogesterone. In contrast, ERα expression in cultured cells was significantly suppressed by all three hormones (P < 0.0001). In control mice or mice treated with a single or double dose of DHT, a dose-dependent increase in body weight was observed (control 22 ± 2 g, single dose 24 ± 2 g, double dose 26 ± 2 g; P = 0.0002). Intracarotid stiffness measured via pulse wave velocity showed a more than two-fold increase in both DHT-treated groups (control 1.9 ± 0.3 m/s, single dose 4.3 ± 0.8 m/s, double dose 4.8 ± 1.0 m/s). This increase in arterial stiffness occurred independent of changes in blood pressure (P = 0.59). Histological analysis of aortic sections using Masson’s trichrome showed a significant decrease in collagen between the control group (24 ± 5%) and the double dose group (17 ± 3%, P = 0.007), despite no changes in aortic wall thickness or smooth muscle content. Lastly, ddPCR showed that in vivo DHT treatment decreased aortic expression of both GPER (control 20 ± 5, single dose 10.5 ± 5.6, double dose 10 ± 4 copies/ng; P = 0.001) and ERα (control 54 ± 2, single dose 24 ± 13, and double dose 23 ± 12 copies/ng; P = 0.003).

**Conclusions:**

These findings indicate that androgen promotes arterial stiffening and cardiovascular damage in female mice and is associated with decreased estrogen receptor expression. These data are important for transgender men, women using testosterone for fitness or reduced libido, as well as patients with polycystic ovarian syndrome.

## Plain language summary

The current study investigated the impact of other hormones on estrogen receptor expression and its impact on vascular health. In both cultured vascular cells and in vivo vascular tissue, dihydrotestosterone decreased the expression of estrogen receptors. Female mice treated with dihydrotestosterone also displayed increased body weight and arterial stiffness despite no change in blood pressure. These findings indicate that increases in testosterone may impact vascular health, which may be important clinically for transgender men, women using testosterone for fitness or reduced libido, as well as patients with polycystic ovarian syndrome.

## Highlights

- Treatment of cultured vascular smooth muscle cells with other hormones, including dihydrotestoterone, significantly decreased the expression of estrogen receptors.
- In vivo treatment of female mice with dihydrotestosterone also reduced vascular estrogen receptor expression and was associated with increased arterial stiffness but not blood pressure.
- These results highlight the need to consider the hormonal milieu in when considering the impact of hormonal changes on vascular health.

## Background

Cardiovascular disease (CVD) is the leading cause of mortality worldwide and is sexually dimorphic in not only incidence but in presentation and diagnosis [1]. While many variables contribute to CVD including genetics, age, activity level, diet, substance use, and sleep, it is well known that sex hormones play a critical role in sex differences in CVD [2, 3]. While many studies focus on the role of estrogen in females and testosterone in males, these steroid hormones differ by only one enzymatic step and are present in the circulation of both sexes. In fact, throughout the female lifespan levels of circulating testosterone are higher than estradiol, although still lower than testosterone levels in men [4]. The role of sex hormones becomes even more important when considering that aging dramatically changes levels of both estrogen and testosterone [5, 6]. How these hormonal changes impact the progression of CVD is still not fully understood.

In female-to-male transgender patients, some studies find no increase in cardiovascular risk factors while others show increases in arterial stiffness and carotid intima-media thickness [7–12]. Disparate findings on the impact of testosterone on arterial remodeling in men versus women also indicates a likely interaction with biological sex [13, 14]. Sex hormone levels are also disrupted in certain endocrine disorders such as polycystic ovarian syndrome (PCOS), which affects 20% of women of reproductive age worldwide [15]. Because of the hyperandrogenism often associated with PCOS, women with the disorder can experience stereotypical symptoms such as increased acne and body hair, infertility, and weight gain [16]. Women suffering from PCOS are also at higher risk of developing a variety of comorbidities including hypertension, diabetes, arterial stiffness, and atherosclerosis [17]. Androgen therapy in transgender men also increases the incidence of PCOS, which in turn promotes these same cardiometabolic disturbances including arterial stiffness [17, 18]. While some lifestyle and pharmacological approaches can reduce risk, additional knowledge is needed on the molecular mechanisms that underlie how changes in or treatment with sex hormones impact vascular health.

Our previous work shows an important role for the novel G protein-coupled estrogen receptor (GPER) in both hypertension and arterial stiffening, both strong risk factors for cardiovascular mortality [19]. We previously showed that estrogen is necessary for female protection from angiotensin II-dependent hypertension in a transgenic rat model, while selective activation of GPER provides the same antihypertensive effect as nonselective estradiol treatment [20, 21]. Moreover, we find that genetic deletion of GPER in mice induces arterial stiffness [22–24]. Considering these findings, we hypothesized in the current study that testosterone may counteract the protective effects of estradiol via GPER on arterial stiffness due to regulation of vascular receptor expression. We utilized dihydrotestosterone (DHT) to avoid aromatization to estrogen and direct binding to GPER as well as the nuclear estrogen receptors alpha (ERα) and beta (ERβ). We determined the impact of DHT treatment both in vitro and in vivo on the vascular expression of GPER and ERα, which we previously showed were the dominant receptor subtypes in aortic tissue [25, 26].

## Methods

### Cell culture

Mouse aortic smooth muscle cells (MOVAS; ATCC CRL-2797) or rat aortic smooth muscle cells (A7r5; ATCC CRL-1444) were grown in 10% FBS media to 80% confluency and then starved for 24 h in charcoal-stripped 0.5% FBS. The plates were treated for 24 h with 100 nm of dexamethasone (DEX), dihydrotestosterone (DHT), or medroxyprogesterone acetate (MPA) or vehicle. After treatment, RNA was extracted, and droplet digital PCR (ddPCR) was used to quantify the expression of both GPER and ERα.

### Karyotyping

Cells were harvested when they reached 75% confluency. Sixty µl of colcemid (10 µg/ml, Life Technologies) was added to each flask containing 10 ml culture medium and incubated at 37℃ for 90 min. Subsequently, cells were treated with 5 ml of 0.05% Trypsin–EDTA for 5 min at 37℃, suspended by mitotic shake-off. Trypsin treatment was stopped by adding 10 ml of 10% FBS culture medium, and suspended cells were pelleted by centrifugation. The cell pellet was resuspended in pre-warmed hypotonic solution (1:1 of 0.8% Na Citrate: 0.075 M KCl) and incubated at 37 °C for 10 min. Then the cells were prefixed with 1 ml of fixative solution (3:1 methanol: acetic acid), spun down and followed by 45 min fixation at room temperature. Air dried chromosome spreads were dropped and treated with trypsin and stained with Giemsa [27]. Chromosome spreading images were captured and analyzed through CytoVision software.

### PCR genotyping

Genomic DNA was isolated from A7r5 cells, and 1 µl (50 ng/µl) was added to a PCR reaction mixture consisting of 8.5 µl nuclease-free water, 12.5 µl Go Taq Master Mix (Promega), 0.5 µl forward primer, and 0.5 ul reverse primer. For positive and negative controls, genomic DNA was extracted from male and female rat tail tissues. Nuclease-free water was used as no template control (NTC). PCR amplification and DNA amplicon agarose gel electrophoresis was conducted according to an established protocol using the following primer sequences: forward Kdm5c (X chromosome) 5’-TTTGTACGACTAGGCCCCAC-3’; forward Kdm5d (Y chromosome) 5’-TTGGTGAGATGGCTGATTCC-3’, and reverse for both 5’-CCGCTGCCAAATTCTTTGG-3’ [28].

### Quantitative PCR (qPCR)

Different passages of MOVAS and A7r5 cells were lysed by the addition of lysis buffer containing 2-Mercaptoethanol, and total RNA was extracted using the RNAeasy Plus Mini Kit (Qiagen). The quality and quantity of RNA were confirmed using a NanoDrop 2000 spectrophotometer (Thermo Fisher Scientific). Gene expression of Xist was quantified using the SYBR Green assay (iTaq Universal SYBR Green supermix, Bio-rad) with 50 ng of complementary DNA (iScript Adv cDNA Kit, Bio-rad) on a real-time PCR system (CFX96, Bio-rad). Primers were obtained from IDT as follows: mouse Xist (Mm.PT.58.8416342, forward 5’GAACTACTGCTCCTCCGTTAC-3' and reverse 5'-CACTCTTCACTCCTCTAAATCCA-3', NR_001570); mouse β-actin mouse (Mm.PT.39a.22214843.g, forward 5’-GATT ACTGCTCTGGCTCCTAG-3' and reverse 5'-GACTCATCGTACTCCTGCTTG-3', NM_007393); rat Xist (forward 5’-TGCCTGGATTTAGAGGAG-3’ and reverse 5’-CTCCACCTAGGGATCGTCAA-3’, NR_132635.2); and rat β-actin (Rn.PT.39a.22214838.g, forward 5'-TCACTATCGGCAATGAGCG-3' and reverse R: 5'-GGCATAGAGGTCTTTACGGATG-3', NM_031144). The rat Xist primer was custom and based on a previous publication, and PCR conditions and cycling programs were also used as previously described [29, 30]. Positive controls were female and male aortas from mice or rats. Quantitative values for Xist mRNA expression are represented as ΔCt values to represent the difference between the threshold cycles for Xist and β-actin.

### Animals and drug treatment

All procedures were approved by the Tulane University Animal Care and Use Committee. Female C57BL/6 mice were imported from Jackson Laboratories at 10 weeks of age. At 15–16 weeks of age, mice were randomized to one of three treatments administered via subcutaneous silastic capsule for 4 weeks: control (CON; empty silastic capsule), group I (1 capsule containing 5 mg of DHT), or group II (2 capsules containing 5 mg DHT). The 5 mg dose of DHT was selected based on previous studies showing it produces plasma concentrations of roughly 30 ng/dl which is in the range for adult men (14–77 ng/dl) but higher than average for adult women (2.6–26 ng/dl) [31, 32]. Body weight was recorded prior to capsule implantation, and body weight, uterine weight, and heart weight was recorded post-euthanasia. Aortas were halved and preserved in either RNALater (top half) for RNA extraction and droplet digital PCR or formalin (bottom half) for aortic morphology and Masson’s trichrome (MTC) staining. Blood was collected by cardiac puncture and subjected to LC–MS at the Endocrine Technologies Core at Oregon Health Sciences University.

### Cardiovascular measurements

Blood pressure was obtained via tail cuff plethysmography using the Kent Scientific CODA Volume Pressure Recording system. Blood pressure was measured at baseline before the initiation of treatment and at weekly intervals during treatment. Each mouse received two training days before testing day. No anesthesia was used on any training or testing day. Within each session, 15 cycles were obtained with the first 5 considered acclimation. The last 10 readings were used to compute the mean as long as tail blood volume was a minimum of 20 µl and the pressure curve was the appropriate shape. Arterial stiffness was assessed via intracarotid pulse wave velocity using a VisualSonics Vevo 1100 high-frequency ultrasound system with MS400 (18–38 mHz) linear probe. As previously described, the carotid artery was imaged in B-mode from the aortic arch to the bifurcation and arrival of the pulse wave was measured in Doppler mode [23].

### Droplet digital PCR (ddPCR)

Aortas were stored in RNAlater before being mechanically homogenized and extracted using the Rneasy Micro Kit (Qiagen #74004). Validated rat and mouse primers for GPER and ERα along with the protocol for ddPCR were previously described [25, 26].

### Aortic histology

Aortas were fixed in formalin for 24 h before being transferred to 70% ethanol, formalin-embedded, and sectioned onto slides. Masson’s Trichrome staining (Newcomer Supply #1403) was carried out according to the manufacturer’s protocol. Image-J was used for analysis of geometry and area fraction of staining for smooth muscle and collagen.

### Statistical analysis

Cell culture experiments in MOVAS cells and all data from the in vivo study were analyzed by one-way ANOVA with Dunnett’s multiple comparisons test. Data from A7r5 cells was analyzed using two-way ANOVA with Sidak’s post-hoc tests.

## Results

**Fig. 1:**
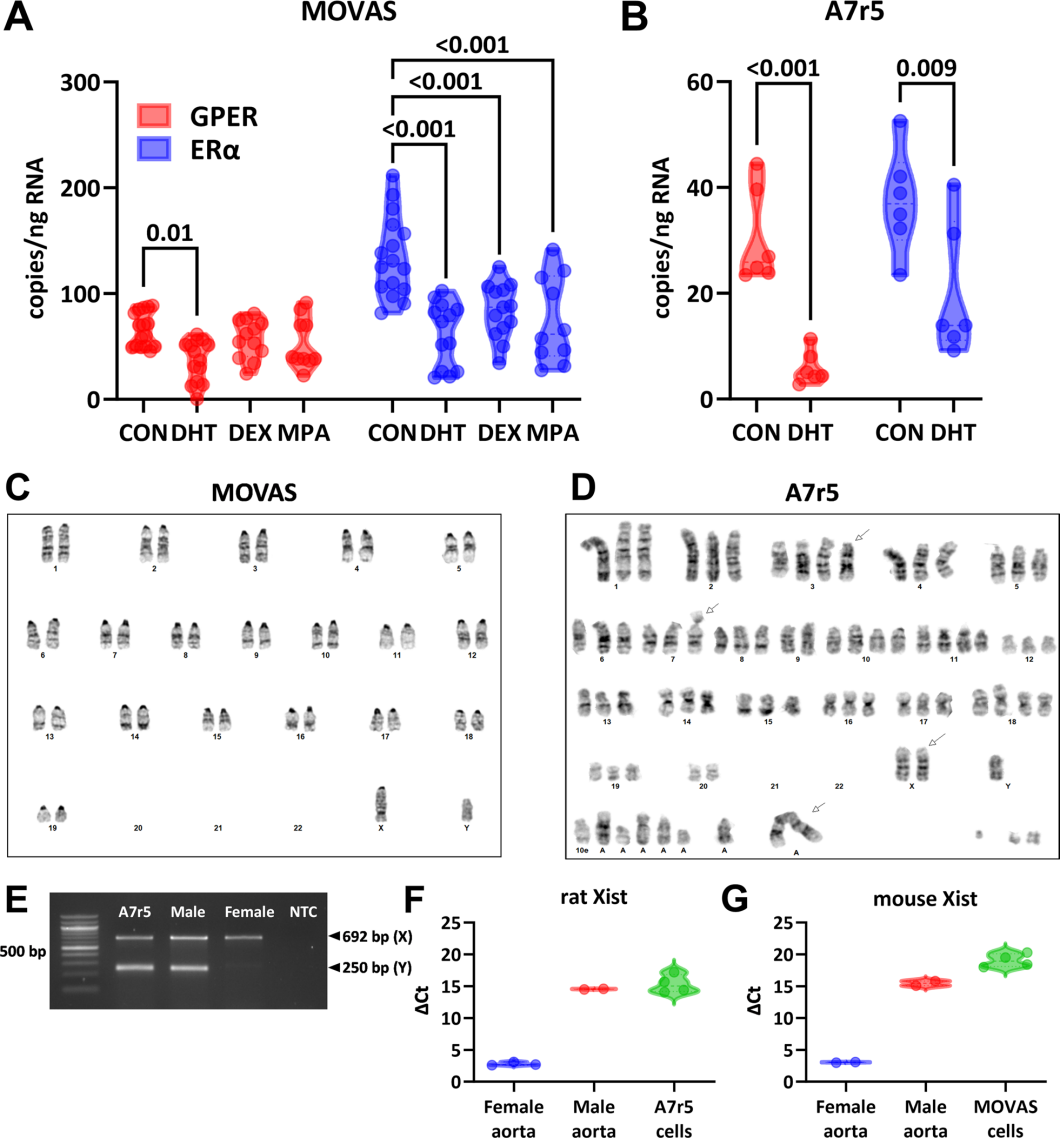
Impact of in vitro hormones on estrogen receptor expression in vascular smooth muscle cells. **A** In mouse aortic smooth muscle cells (MOVAS), GPER expression was significantly decreased in comparison with DMSO vehicle (CON) by dihydrotestosterone (DHT) (One-way ANOVA, P = 0.002; Dunnett’s post-hoc test, N = 10–16). ERα was significantly downregulated by DHT, dexamethasone (DEX), and medroxyprogesterone acetate (MPA; One-way ANOVA, P < 0.0001; Dunnett’s post-hoc test, N = 10–16). **B** In the A7r5 rat aortic smooth muscle cell line, DHT significantly supressed mRNA for both GPER and ERα (Two-way ANOVA, P < 0.0001, Sidak’s post-hoc test, N = 6). Karyotype of **C** MOVAS and **D** A7r5. **E** PCR genotyping of A7r5 cells shows evidence of male genotype (XY). qPCR data for **F** rat and **G** mouse Xist represented as the difference between the threshold cycles for Xist and β-actin (ΔCt)

Expression of GPER in mouse vascular smooth muscle cells (MOVAS) was significantly decreased by DHT (66 ± 4 vs. 37 ± 5 copies/ng RNA, P = 0.0004, n = 12–14) but neither DEX (57 ± 5 copies/ng RNA, P = 0.49, n = 12) nor MPA (53 ± 8 copies/ng RNA, P = 0.25, n = 10; Fig. 1A). In contrast, ERα mRNA was decreased by DEX (135 ± 9 vs. 84 ± 7 copies/ng RNA, P = 0.0006, n = 13–16), DHT (62 ± 8 copies/ng RNA, P < 0.0001, n = 13), and MPA (75 ± 13 copies/ng RNA, P = 0.0002, n = 10). The impact of DHT on GPER and ERα was also tested in the A7r5 rat aortic smooth muscle cell line. Again, DHT induced significant downregulation of both GPER (31 ± 4 vs. 6 ± 1 copies/ng RNA, P = 0.0004, n = 6) and ERα (37 ± 4 vs. 20 ± 5 copies/ng RNA, P = 0.009, n = 6; Fig. 1B). Gene expression of ERβ was not tested because we previously showed it was below the level of detection in both rat and mouse aorta [25, 26].

Since there were major differences in the absolute copy number for GPER and ERα, we went on to determine whether these two cell lines were of male, female, or mixed sex origin. Karyotyping of the MOVAS cell line showed evidence of an Y chromosome, indicating that at least some cells were of male origin (Fig. 1C). A7r5 karyotyping showed near tetraploidy with complex chromosomal rearrangements and several unidentified marker chromosomes (Fig. 1D), which has previously been reported [33]. Moreover, we identified an incomplete Y chromosome, segments of which may have been present within the unidentified marker chromosomes. Therefore, PCR genotyping was performed on the A7r5 cell lines to provide additional evidence for the presence of Y chromosome genes in this cell line (Fig. 1E). To exclude the possibility that female cells were also present, we probed for Xist RNA, which is only expressed in the presence of two X chromosomes. Xist was not detected in either cell line, providing evidence that both MOVAS and A7r5 cell lines are of male origin.

**Fig. 2:**
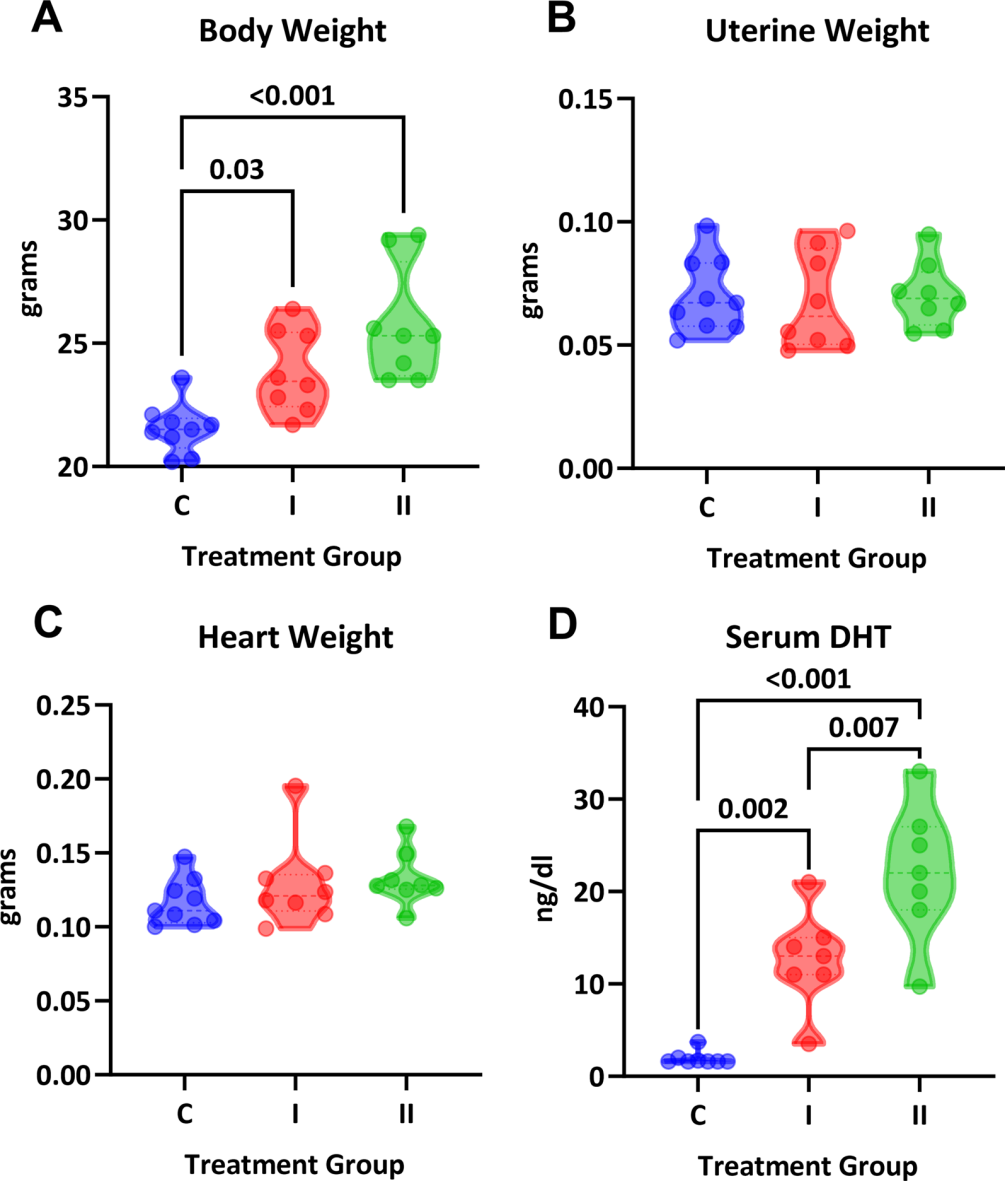
Physiological parameters in female mice treated with DHT. Intact female mice were randomized to three treatment groups: control (C), one capsule of DHT (I) or two capsules of DHT (II). **A** DHT increased body weight in a dose-dependent manner (One-way ANOVA, P = 0.0002; Sidak’s post-hoc test, N = 8–9). Neither **B** uterine weight or **C** heart weight were different across group (One-way ANOVA, P > 0.05). **D** Serum concentrations of DHT showed statistical significance for linear trend (P = 0.04)

**Fig. 3:**
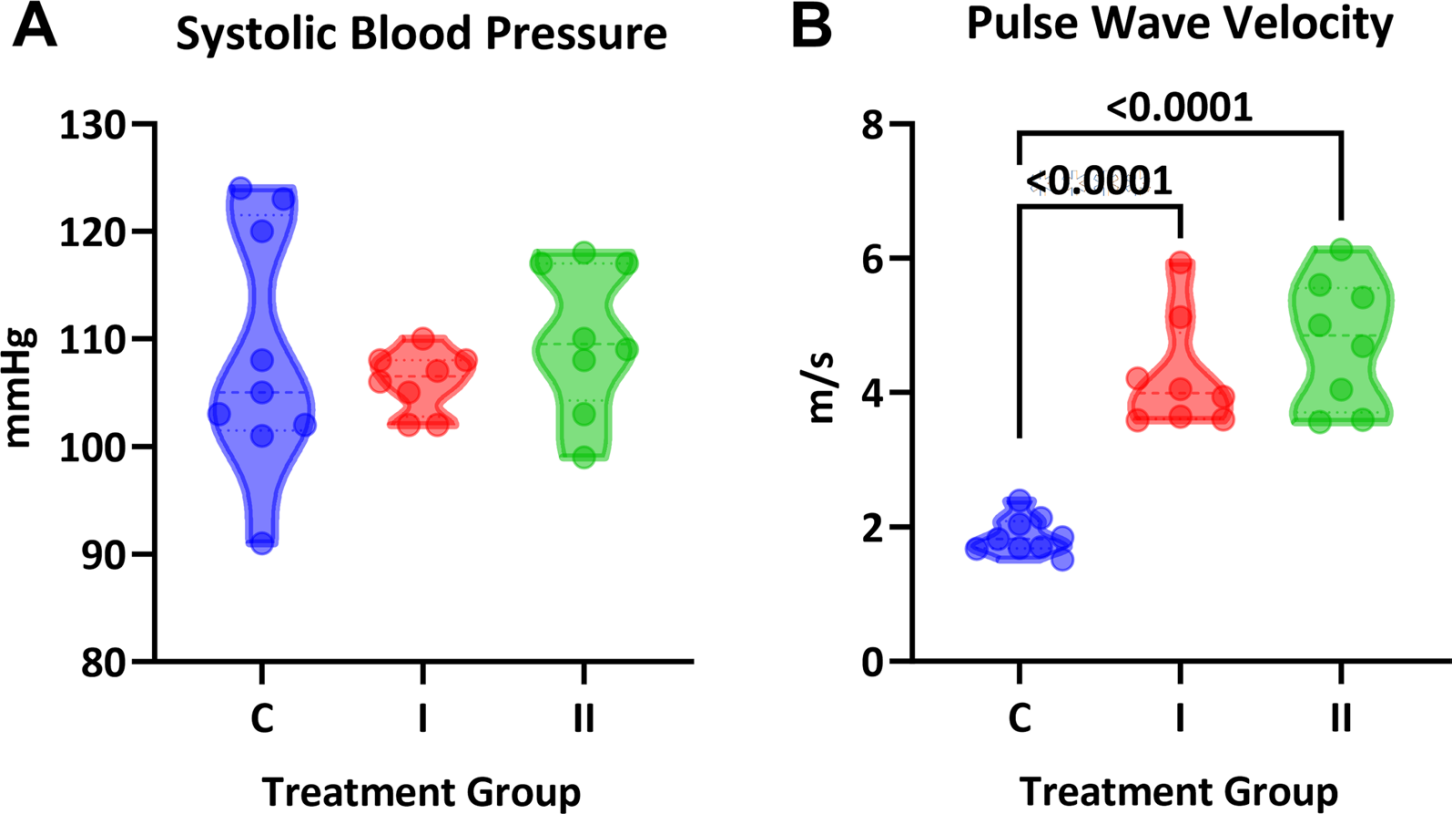
Cardiovascular parameters in female mice treated with DHT. **A** Systolic blood pressure was similar across groups (One-way ANOVA, P = 0.59). **B** Pulse wave velocity was significantly increased in both DHT-treated groups (One-way ANOVA, P < 0.0001, Dunnett’s post-hoc test)

**Fig. 4:**
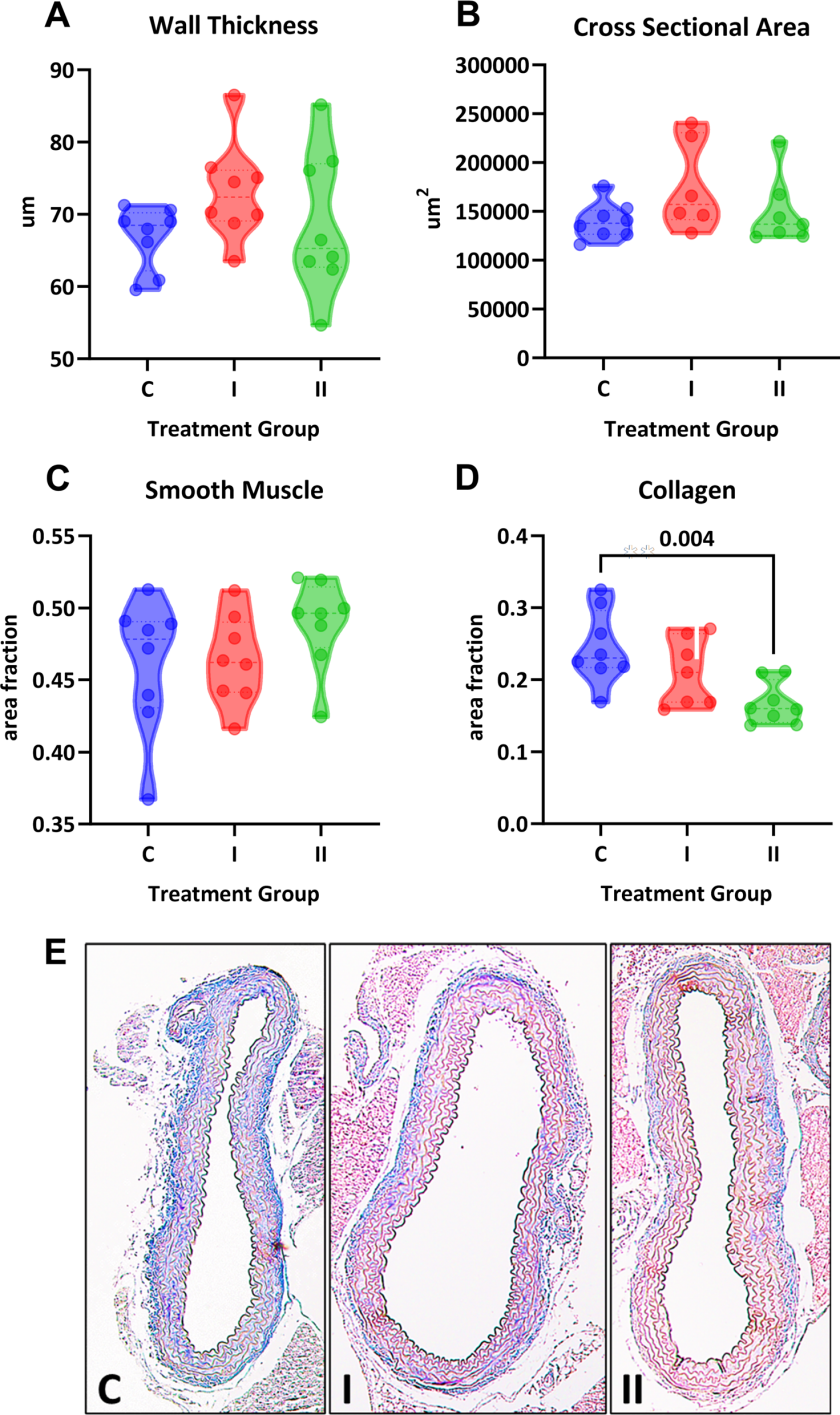
Histological analysis of aortic cross-sections. **A** Aortic wall thickness, **B** aortic cross-sectional area, and **C** smooth muscle content were not different across groups (One-way ANOVA, P > 0.05). **D** Collagen content was significantly decreased in the high dose DHT group (One-way ANOVA, P = 0.007, Dunnett’s post-hoc test). **E** Representative aortic cross sections from each treatment group stained with Masson’s Trichrome

In vivo treatment with DHT increased body weight in a dose-dependent manner but did not impact heart or uterine weight (Fig. 2A-C). Serum DHT measured by LC–MS showed a significant stepwise increase in values (one-way ANOVA, P < 0.001; Fig. 2D). Serum estradiol was below the lower limit of detection by LC–MS. Systolic blood pressure was not significantly different between groups and throughout the study (one-way ANOVA, P = 0.59; Fig. 3A). Intracarotid pulse wave velocity, a measure of arterial stiffness, was twice as high in both treatment groups compared with the control group (1.9 ± 0.09, 4.3 ± 0.3, and 4.8 ± 0.3 m/s; P < 0.0001; Fig. 3B). To assess whether the arterial stiffness was associated with changes in vascular fibrosis, aortic sections were stained with Masson’s Trichrome (Fig. 4E). No differences were observed in aortic wall thickness (P = 0.24; Fig. 4A) or cross-sectional area (P = 0.18; Fig. 4B). Smooth muscle content was also not impacted by DHT treatment (P = 0.26; Fig. 4C). Surprisingly, we found significantly lower vascular collagen content at the high dose of DHT (P = 0.003; Fig. 4D).

**Fig. 5:**
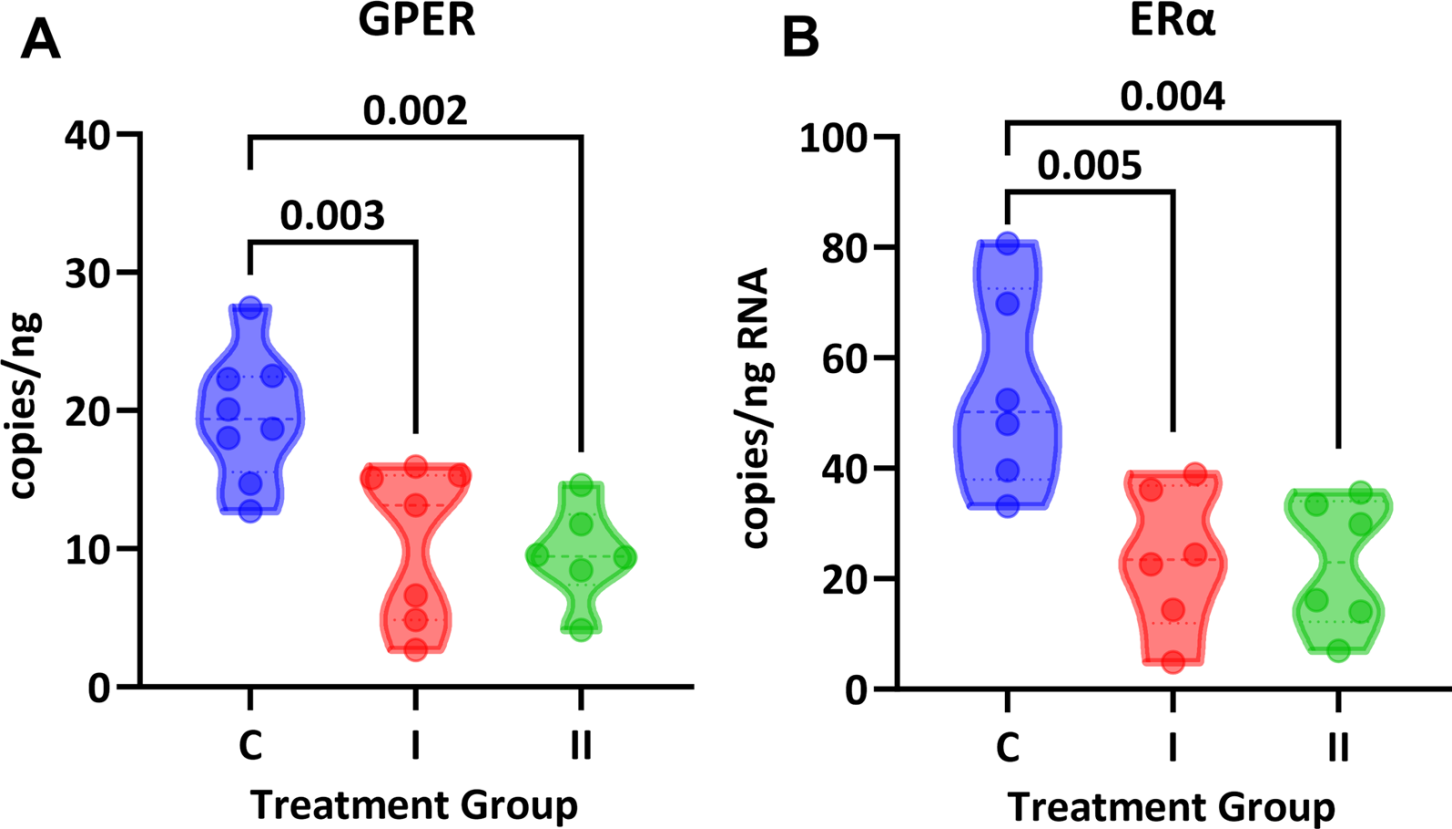
RNA analysis of estrogen receptors in aorta. Both **A** GPER and **B** ERα were significantly downregulated in aortic tissue from both DHT-treated groups (One-way ANOVA, P < 0.01, Dunnett’s post-hoc test)

Lastly, vascular estrogen receptor mRNA was analyzed in aortic samples. Both DHT treatment groups showed lower copy numbers for both GPER and ERα in comparison with controls (Fig. 5). GPER was decreased from 20 ± 2 copies/ng RNA in controls to 11 ± 2 copies (P = 0.003) in the low dose group and 10 ± 1 copies/ng RNA (P = 0.002) in the high dose group. ERα levels were at 54 ± 7 copies/ng RNA in the control group and decreased to 24 ± 5 (P = 0.005) and 23 ± 5 (P = 0.004) in the I and II capsule groups, respectively.

## Discussion

The current study showed that both in vitro and in vivo treatment with DHT downregulates vascular expression of GPER and ERα. Moreover, we found that this is associated with increased arterial stiffness in adult female mice. This study confirms that crosstalk or feedback loops exist between different sex hormones so that altered levels of one hormone may impact signaling of another and underscores the importance of assessing the impact of hormones together rather than separately. Taken with our previous work showing that GPER deletion increases vascular stiffness, these data underscore the importance of GPER signaling on vascular health. Additional work is needed to fully elucidate the specific cellular mechanisms involved in GPER’s protection from vascular stiffening.

In cultured vascular smooth muscle cells, DHT decreased the expression of GPER, while DEX, DHT, and MPA downregulated ERα. These results suggest that ERα levels are sensitive to androgens, glucocorticoids, and progestins in vascular smooth while GPER is only sensitive to androgens. The ability of progestins to downregulate estrogen receptors in the endometrium is well known and opposes both endometrial proliferation and carcinogenesis in response to unopposed estrogen [34, 35]. The relationship between glucocorticoids such as dexamethasone and estrogen receptor signaling is less clear, with most studies showing an ability of DEX to inhibit downstream signaling without direct effects on receptor expression [36, 37]. Previous work in prostate cancer cells similarly showed DHT-induced suppression of GPER that occurs through androgen receptor binding to transcription factors Sp1 and Sp3 and prevent their transcription of GPER [38, 39]. Reciprocal interference between the gene transcription outputs for estrogen versus androgen is also demonstrated in breast cancer [40]. We propose that androgen-induced downregulation of estrogen receptor mRNA lessens the beneficial effects of estrogen on cardiovascular function in both sexes.

We found that both low and high dose DHT for 4 weeks doubled intracarotid stiffness assessed via PWV in otherwise healthy, adult female mice. PWV is a strong predictor of cardiovascular mortality in patients independent of other traditional cardiovascular risk factors [41]. Whether the DHT-induced arterial stiffening in this study was tied to the increased body mass induced by DHT cannot be excluded. In fact, arterial stiffness is also induced in mice by a high fat diet and similarly occurs in the absence of an increase in blood pressure [42]. While lifestyle interventions such as avoiding smoking, following a healthy diet, and exercising can reduce CVD risk, there is an increased risk secondary to hyperandrogenism because collagen dysregulation may happen independently of weight gain.

In the current study, DHT treatment in 15-week-old female mice for 4 weeks did not impact blood pressure. In contrast, DHT treatment initiated in female Sprague–Dawley rats at 5 weeks of age resulted in elevated mean arterial pressure by 12 weeks of treatment [43]. The difference in initiation time is most likely not the reason for this discrepancy, as a study by the same group starting DHT post-puberty at 7 weeks of age also showed an increase in blood pressure [44]. Therefore, the discrepancy in blood pressure response between the current study and previous studies in rats could be due to either differences in treatment time or species differences in steroid metabolism [45]. Future studies extending our DHT treatment protocol in mice to 12 weeks will be able to ascertain whether arterial stiffness *precedes* hypertension. We also did not observe evidence of cardiac hypertrophy in our study, although testosterone treatment for 11 weeks in ovariectomized female rats increased both blood pressure as well as cardiac hypertrophy [46]. Since our previous work shows that arterial stiffness precedes other indicators of cardiovascular damage in otherwise healthy mice, androgen exposure for longer periods would be expected to increase other indices such as hypertension and cardiac remodeling.

The increased arterial stiffness in the current study was not linked to changes in vascular geometry or smooth muscle content but was associated with a decrease in collagen. Dysregulation of collagen is often linked to aging-induced arterial stiffness, but these studies commonly indicate that an *increase* in vascular collagen deposition is the culprit [47, 48]. Both in the current study and in our previous work, collagen does not increase along with PWV and instead many times decreases [23, 24, 49]. This discrepancy may indicate the presence of significant sex differences in the mechanisms of arterial stiffening. Changes in the composition and interaction of extracellular components such as elastin fragmentation, collagen fiber type, or matrix crosslinking may underlie the arterial stiffening that occurred with DHT treatment [50]. The role of vascular smooth muscle should not be ignored, as both the stiffness of the smooth muscle cell as well as its adhesion to the matrix can increase arterial stiffness in the absence of changes in the extracellular matrix [51]. More studies are needed to identify the specific vascular mechanisms that cause arterial stiffening in females in response to elevated DHT.

DHT values were 1.9, 12.6, and 22.1 ng/dl for control, one capsule, and two capsule groups, respectively, recapitulating levels at the lower range in adult men (14–77 ng/dl) but high end for adult women (2.6–26 ng/dl) [18, 19]. E2 levels were below the level of detection by LC–MS, but since uterine weight is an extremely sensitive measure of circulating estradiol and no differences were seen between groups, DHT did not likely affect circulating estradiol. Historically, mouse estradiol levels were based on immunoassays, but more recent work shows that these assays are inaccurate [52, 53]. While the measurement of serum estradiol by LC–MS is considered the gold standard for clinical purposes [54–56], for mouse serum the levels are near or below the lower limit of detection with the current technology available [57].

A review of clinical data on the effects of testosterone administration in female-to-male transgender patients does not show a significant increase in overall cardiovascular events [7]. While a recent systematic review highlights mixed results, some individual studies find that androgen therapy in transitioning patients significantly increases arterial stiffness as well as carotid intima-media thickness [10–12]. Similarly, hormone therapy in the female-to-male transgender population increases the incidence of PCOS, resulting in a higher risk for obesity and associated cardiometabolic disturbances [18]. The impact of testosterone on arterial remodeling is most likely impacted by biological sex as well as the presence or absence of other circulating hormones [13, 14]. DHT was our androgen of choice for inducing hyperadrenergic conditions in female mice because it cannot be aromatized into estrogen like testosterone, allowing a higher degree of control over hormonal effects in our mice. While DHT alone is not used clinically to diagnose PCOS, the testosterone to DHT ratio has been proposed as an indicator of a stronger PCOS phenotype [58]. In addition, this method of DHT treatment induces a PCOS phenotype in C57BL/6 J female mice as evidenced by irregular cycles, ovulatory dysfunction, polycystic ovary morphology, increased body weight, and impaired fasting glucose [59]. While the direct translation of this treatment regimen to clinical conditions may be limited, the main goal of our study was to pioneer new findings for the physiological impact of enhanced androgen receptor activation in female cardiovascular health.

## Conclusions

This study is the first to show an impact of androgens on arterial stiffening in female mice and has important implications for not only PCOS patients but also women taking androgens for fitness, gender transitioning, or reduced libido. The most recent clinical guidelines recommend against prescribing androgen therapy to women for cardiovascular, metabolic, or general well-being and only indicates its use for hypoactive sexual desire disorder [60]. The rationale for these recommendations is mostly due to a lack of well-defined guidelines and symptoms for these other uses along with a lack of data on the efficacy and safety in otherwise healthy women. Along with the findings from the current study, this indicates an important need for further research on the long-term vascular effects of testosterone treatment in women as well as strategies to protect these patients.

## Data Availability

The datasets used during the current study are available from the corresponding author on reasonable request.

## Abbreviations

ddPCR: Droplet digital PCR
DXA: Dexamethasone
DHT: Dihydrotestosterone
ERα: Estrogen receptor alpha
GPER: G protein-coupled estrogen receptor
MPA: Medroxyprogesterone acetate
PCOS: Polycystic ovarian syndrome
